# Rickets

**Published:** 1895-10

**Authors:** Charles S. Shaw

**Affiliations:** Pittsburg, Pa.


### RICKETS.*

By CHARLES S. SHAW, M.D.,
PITTSBURG, PA.

Rickets may be defined as a constitutional disease of infancy and early childhood affecting the whole body, but chiefly characterized by a softening and deformity of the osseous system.

Since the publication of Glisson's treatise in 1650, rachitis, or rickets, has received the attention of so many observers that it now has a very extensive bibliography of its own; but in spite of the large number of investigators there is much concerning the disease that is yet obscure. In this country rickets has not, till recently, received much notice, for the reason that it has become common in the United States only in recent years. The cause of its noticeable increase is increased immigration from European countries where it prevails, and the distribution through the Northern cities of the American negro which followed the abolition of slavery and the close of the civil war.

*Read before the Allegheny County Medical Society, June 18, 1895.

The geographical distribution of rickets is not its least interesting feature. It is practically unknown in tropical countries, and is a disease peculiar to the northern temperate zone. But it also has racial preferences. Kassovitz is authority for the statement that eighty per cent. of the children of Vienna are rachitic. It is also very common in northern Italy, France, Germany, Russia, and Great Britain; but, singularly, it is rare among the Irish. It is rare, too, in Australia, and essentially absent in China and Japan. The American negro is very subject to rickets; it may be said that every Northern negro child is more or less rachitic. On the other hand, the native African does not suffer from the disease.

It is these peculiar characteristics in geographical and racial distribution that obscures to a degree the etiology of the disease. The common and doubtless correct opinion at present ascribes it to malnutrition in the broadest sense of the term. Hence any condition that produces malnutrition may be causative of rickets. This includes not only improper and insufficient food (which probably is the greatest agent in its production), foul air, and general unhygienic surroundings, but also congenital weakness and cachexias, stomachic and intestinal maladies, and all wasting diseases.

The view of Parrot, that rickets is always a manifestation of congenital syphilis, is untenable. Syphilis, it is true, may cause rickets by producing the state of malnutrition favorable to the development of the disease, but it has no specific influence. Vogel's opinion, that the disease always is caused by impure air, is also entirely too restricted. Microli, of Naples, claims that the disease is due to pyogenic organisms, and that he has found the staphylococcus and the streptococcus in rachitic bone tissue. Chambier, of Tours, believes it to be due to a specific germ, and that it is contagious and endemic. It is impossible to reconcile these views to the universally observed facts of the disease.

There is another erroneous opinion extant concerning the social status of the rachitic child. The disease is most common among the poor, for with them the conditions that favor its advent most abound; but it is not as rare among the well-to-do as common opinion places it. Malnutrition and its follower rickets may and do occur in the children of all classes. The greater number of the cases occur in large cities, particularly manufacturing centers, for obvious reasons; but it also appears occasionally in suburban and rural children. I can find no reason why the Irish are exempt, for the habits and environment of this people do not differ from those of other nationalities to a degree sufficient to explain their immunity. Neither do I know why the American negro should be so universally affected. Whatever may be his other sanitary sins, our colored brother always takes good care of his stomach, and is probably better fed than most others of his social status; so the commonest accepted cause of the disease, improper and insufficient food, does not generally hold with him.

The absence of the disease among the Japanese is credited to the unusual cleanliness of that people, the universal nursing of the children by their mothers, and the free use of oils and fish as foods. The rarity of the disease in Australia is doubtless due, as it previously was in America, to the robust constitutions, active outdoor lives, and salubrious surroundings of the colonists. The freedom of tropical countries from the disease is attributed to the outdoor life of the people. The far north derives its immunity from the oleaginous diet of the inhabitants.

While all the tissues of the body are more or less affected in rickets, it is upon the bones that the most serious and permanent injury falls. The diseased process may be briefly described as an abnormal elaboration of the cartilaginous or organic part of the bone with a delayed or absent ossification. The normal proportion of earthy to mineral parts in bone, one to two, is sometimes reversed, the organic part being twice as great as the inorganic. The epiphyses of the long bones take on an unusually active cartilaginous growth, which results in an enlargement at the extremities; at the same time the bone grows in thickness through the deposit of cartilaginous cells under the periosteum. The usual absorption of bony substances within the medullary canal, which is a step in the normal growth of bone, continues till, in pronounced cases, all firmness is lost, and the bone may be cut through with a knife with ease. Essentially the same process takes place in the flat bones. As recovery occurs the earthy or normal deposit replaces the gelatinous substance and the bone becomes firm, though it retains to some degree its unnatural shape and size. Unusual strength of limb in adult life is not impossible with infantile rickets.

The bones of the cranium are always more or less affected. The edges of these bones, which correspond to the epiphyses of the long bones, take on the cartilaginous growth, they become thickened and the fontanelles remain open. The cranium becomes enlarged and approximates a cubical shape from the prominence of the frontal eminences and the centers of the parietal bones. As the rachitic process advances the bones become soft (craniotabes), and in severe cases may be so deficient in firmness as to permit indentation by the fingers. The spinal column is relaxed and the child, if able to sit up, leans forward, simulating kyphosis. The scapulæ do not seem to be much affected, but the clavicles are always thickend and usually bent, the deformity being an exaggeration of the normal curves. The ribs show a beading at the costo-condral junctions, the so-called "rachitic rosary." The deformity known as pigeon breast is one of the common results of rickets. The muscular action during respiration, and the atmospheric pressure, draws the softened ends of the ribs inward, producing a bilateral groove shaped like an inverted v, with its apex at the top of the sternum. The horizontal depression, Harrison's groove, corresponds to the insertion of the diaphragm, and is caused by the action of that muscle.

Of equal importance is the deformity of the bony pelvis, which, in after life, in females, is a source of suffering and danger. The sacrum projects forward because of the weight of the spinal column, and the weight of the body on the femurs when standing, and on the tuber ischii when sitting, pushes the sides of the pelvis inward so that a section shows a trefoil outline instead of the normal cordate shape.

In the upper extremity the humerus is thickened and curved at about the insertion of the deltoid muscle. The lower end of the radius is also thickened and curved. The deformity of the femur, like that of some other long bones, is usually an exaggeration of its natural curve. The rachitic tibia is bowed forward about the upper part of the lower third. This deformity is the result both of the weight of the foot as the child lies on its back, and the action of the muscles of the calf.

With these changes in the bony skeleton is a relaxation of the ligaments, and a general flabbiness and want of tone in the muscles. The lungs are compressed and contracted, and the heart hypertrophied. The liver and the spleen are occasionally enlarged.

The symptoms of rickets vary with the stage of the disease. Before the appearance of the changes in the bones, the infant is peevish and cross. It lies quietly upon its back and evidently dreads to be moved. It cries out when taken up, and will often throw off the bed clothes as though the pressure of them was painful. Though emaciation is not common, there is a general feebleness noted. The head sweats profusely, often soaking the pillow. the appetite is capricious and the sleep is broken. Evidences of gastro-intestinal catarrh are present; and bronchial irritation usually exists. Doubtless not a few cases of rickets are overlooked by the physician because the bronchial or intestinal malady masks the underlying cause of the illness.

Very soon evidences of bone involvement appear. The English and continental writers all note the invariable and early beading of the ribs. In my experience the rachitic rosary is not so constantly present, the first sign of bone disease appearing in the radius and tibia. In this country, at least, craniotabes is one of the infrequent conditions.

The same may be said of the neuroses which accompany rickets. While it is probably true that rickety children are more apt to have general convulsions than healthy ones, I believe the liability is greatly exaggerated, the proportion who suffer is small, and in these the spasm may usually be attributed to gastro-intestinal irritation and the absorption of toxines. Neither have I found the dangerous neurotic complication, lyringismus stridulus; in fact, the only cases of this spasm I have seen were in children in which rickets did not exist. It must be that craniotabes, general convulsions, and laryngismus stridulus only appear in advanced cases, and these are not at all common in America.

One of the invariable symptoms is delayed dentition. The teeth are slow in making their appearance; but, as Carpenter has pointed out, when they do come, they are not imperfect and prone to early decay, as is commonly stated, but are usually sound and regular.

The protuberance of the abdomen, and the detection of the lower border of the liver lower down than normal, has led to a common but erroneous opinion that the liver is always enlarged. This organ is affected in but a small proportion of cases. The contraction of the chest pushes the liver lower down in the abdomen, and the general lack of muscular tonicity favors its ready descent. The protuberant belly is also due to the flabby muscles, and to the presence of gas in the intestines. The production of gas is favored by the intestinal indigestion, and its retention is due to the feeble and relaxed muscular coat of the bowel.

Presuming the cause of rickets to be malnutrition, the indications for both its prevention and cure are evident. Pure air, cleanliness, and especially proper food are essential. Most cases are due to defective diet, and the defect is usually a lack of fat in the food. If the child is nursed at the breast, the mother's milk will be found to be lacking in fatty elements; if it is artificially fed, the same want will be demonstrable.

Of medicinal agents, cod liver oil holds the first place. When it cannot be borne by the stomach, it may be used by inunction with the utmost satisfaction. Indeed, the rubbing and massage incident to this use of the oil is of distinct value. Olive oil or cocoa butter may be substituted for the cod liver oil in inunction, and in my experience these have been equally of value. Kassovitz claims a specific power for phosphorus, which he gives in doses of about 1-125th of a grain dissolved in olive oil.

The correction of deformities in after-life is often feasible by operative measures, but these should be deferred as long as possible, for there is a constant tendency during growth toward natural correction.

---

### TREATMENT OF CHAPPED HANDS.

The following formula is in use in Germany:

| | |
|---|---|
| Menthol | gr. x. |
| Olive oil | gtt. xx. |
| Salol | gr. xx. |
| Lanoline | ℥ iss. |

To be applied to the parts affected twice a day.—*Med. Journal.*

